# Secretion of fibronectin by human pancreatic stellate cells promotes chemoresistance to gemcitabine in pancreatic cancer cells

**DOI:** 10.1186/s12885-019-5803-1

**Published:** 2019-06-17

**Authors:** Manoj Amrutkar, Monica Aasrum, Caroline S. Verbeke, Ivar P. Gladhaug

**Affiliations:** 1 Department of Pharmacology, Institute of Clinical Medicine, University of Oslo, Blindern, 0316 Oslo, Norway; 2 Department of Hepato-Pancreato-Biliary Surgery, Institute of Clinical Medicine, University of Oslo, PO Box 1171, Blindern, 0318 Oslo, Norway; 3 Department of Pathology, Institute of Clinical Medicine, University of Oslo, Blindern, 0316 Oslo, Norway; 4 Department of Pathology, Oslo University Hospital Rikshospitalet, Nydalen, 0424 Oslo, Norway; 5 Department of Hepato-Pancreato-Biliary Surgery, Oslo University Hospital Rikshospitalet, Nydalen, 0424 Oslo, Norway

**Keywords:** Pancreatic stellate cells, Gemcitabine, Fibronectin, Pancreatic cancer, Chemoresistance, Extracellular matrix

## Abstract

**Background:**

Gemcitabine remains a cornerstone in chemotherapy of pancreatic ductal adenocarcinoma (PDAC) despite suboptimal clinical effects that are partly due to the development of chemoresistance. Pancreatic stellate cells (PSCs) of the tumor stroma are known to interact with pancreatic cancer cells (PCCs) and influence the progression of PDAC through a complex network of signaling molecules that involve extracellular matrix (ECM) proteins. To understand tumor-stroma interactions regulating chemosensitivity, the role of PSC-secreted fibronectin (FN) in the development of gemcitabine resistance in PDAC was examined.

**Methods:**

PSC cultures obtained from ten different human PDAC tumors were co-cultured with PCC lines (AsPC-1, BxPC-3, Capan-2, HPAF-II, MIA PaCa-2, PANC-1 and SW-1990) either directly, or indirectly via incubation with PSC-conditioned medium (PSC-CM). Gemcitabine dose response cytotoxicity was determined using MTT based cell viability assays. Protein expression was assessed by western blotting and immunofluorescence. PSC-CM secretome analysis was performed by proteomics-based LC-MS/MS, and FN content in PSC-CM was determined with ELISA. Radiolabeled gemcitabine was used to determine the capacity of PCCs to uptake the drug.

**Results:**

In both direct and indirect co-culture, PSCs induced varying degrees of resistance to the cytotoxic effects of gemcitabine among all cancer cell lines examined. A variable degree of increased phosphorylation of ERK1/2 was observed across all PCC lines upon incubation with PSC-CM, while activation of AKT was not detected. Secretome analysis of PSC-CM identified 796 different proteins, including several ECM-related proteins such as FN and collagens. Soluble FN content in PSC-CM was detected in the range 175–350 ng/ml. Neither FN nor PSC-CM showed any effect on PCC uptake capacity of gemcitabine. PCCs grown on FN-coated surface displayed higher resistance to gemcitabine compared to cells grown on non-coated surface. Furthermore, a FN inhibitor, synthetic Arg-Gly-Asp-Ser (RGDS) peptide significantly inhibited PSC-CM-induced chemoresistance in PCCs via downregulation of ERK1/2 phosphorylation.

**Conclusions:**

The findings of this study suggest that FN secreted by PSCs in the ECM plays a key role in the development of resistance to gemcitabine via activation of ERK1/2. FN-blocking agents added to gemcitabine-based chemotherapy might counteract chemoresistance in PDAC and provide better clinical outcomes.

**Electronic supplementary material:**

The online version of this article (10.1186/s12885-019-5803-1) contains supplementary material, which is available to authorized users.

## Background

Pancreatic ductal adenocarcinoma (PDAC), generally referred to as pancreatic cancer, is a highly malignant disease characterized by late diagnosis, early metastasis, limited response to chemotherapy, and an extremely poor prognosis [1–3]. Despite significant advances in the understanding of the pathobiology of the disease over the past decades, overall 5-year survival rate has only marginally improved to merely 7%, making it the fourth leading cause of cancer-related mortality in the Western world [4]. Moreover, it is projected to rank second by 2030 [5]. While surgery offers the only potential curative treatment, only 15–20% of patients have resectable disease at the time of diagnosis, and these patients also carry the risk of disease recurrence within a year post-surgery [6]. Chemotherapy is therefore a crucial component in the treatment of the disease, both for unresectable (locally advanced or metastatic) disease or in the neoadjuvant and adjuvant settings [7]. Gemcitabine (dFdC) is a nucleoside pyrimidine analogue that has long been the backbone of chemotherapy for PDAC in all stages of the disease [8, 9]. However, gemcitabine has suboptimal clinical outcomes caused by molecular mechanisms limiting its cellular uptake, activation and overall efficacy, as well as by the development of chemoresistance within weeks of treatment initiation [10, 11].

The presence of a dense desmoplastic stroma that constitutes the bulk of the tumor mass is a prominent feature of PDAC [12, 13]. The stroma is composed of several different cell types and various extracellular matrix (ECM) components such as collagens and fibronectin (FN), which provide structural integrity and initiate signaling cascades that promote tumor cell survival, proliferation, and migration [14–16]. The activated pancreatic stellate cell (PSC) – a type of cancer-associated fibroblast (CAF) – is the primary source of ECM components and also the major driver and organizer of the desmoplastic reaction in PDAC [17]. In the healthy pancreas, PSCs exist in a quiescent state, but in response to various pathophysiological stimuli such as injury, inflammation and induction of neoplasia in ductal epithelium, the PSCs are activated [14, 18]. Through the secretion of excessive amounts of ECM components, activated PSCs induce the fibrosis that is characteristically found in PDAC [18, 19].

Several lines of evidence suggest that the tumor stroma contributes to gemcitabine chemoresistance through a range of mechanisms, including impairment of drug delivery due to decreased functional microvascularity, activation of anti-apoptotic pathways, promotion of epithelial-mesenchymal transition, and upregulation of tumor-supporting inflammatory cytokines [10, 20–22]. While the cause of gemcitabine chemoresistance is clearly multifactorial and PSCs are implicated in several of these processes, the precise role of the PSCs in the development of chemoresistance is still not fully understood [23–26]. Previous studies have also indicated that pancreatic cancer cells (PCCs) embedded in collagen gels [27] or cultured on FN-coated dishes [28], show increased resistance to gemcitabine, suggesting the possibility that ECM-induced signals might be involved.

In the present study we hypothesized that secreted soluble factors implicated in gemcitabine chemoresistance in pancreatic cancer may be identified through analysis of the PSC secretome. To this end, we examined conditioned medium from ten primary human PSC cultures for its secretome content and evaluated its effects on PCC’s gemcitabine chemosensitivity in order to explore the effects of ECM proteins on pancreatic cancer chemoresistance.

## Methods

### Reagents

Reagents were purchased from the following sources: Dulbecco’s modified Eagle’s medium containing 4.5 g/l glucose (DMEM), penicillin-streptomycin (Pen-Strep), amphotericin B, Trypsin/EDTA (1X), fetal bovine serum (FBS) and Pierce™ BCA protein assay kit from Thermo Fisher Scientific (Waltham, MA, USA); bovine serum albumin (BSA), Collagen type I solution from rat tail, FN, gemcitabine hydrochloride, 3-(4,5-Dimethylthiazol-2-yl)-2,5-Diphenyltetrazolium Bromide (MTT), phosphate buffered saline (PBS) and human fibronectin ELISA kit from Sigma-Aldrich (St Louis, MO, USA); FN inhibitor (RGDS) from Santa Cruz Biotechnology (Santa Cruz, CA, USA); [^3^H]-gemcitabine from Moravek Biochemicals Inc. (Brea, CA, USA); Ultima Gold from Perkin Elmer (Waltham, MA, USA); PD98059 from Calbiochem (La Jolla, CA); human anti-alpha smooth muscle actin (αSMA; BS66) from Nordic Biosite AB (Taby, Sweden); anti-AKT, anti-phospho-AKT, anti-glial fibrillary acidic protein (GFAP; GA5), anti-epithelial cell adhesion molecule (EpCAM; VU1D9), anti-p44/42 MAPK (ERK1/2), anti-phospho-ERK1/2, anti-vimentin (D21H3) and anti-GAPDH from Cell Signaling Technology (Beverly, MA, USA); anti-phosphoinositol-3-kinases (PI3K) from Merck Life Science AS (Oslo, Norway); secondary HRP-conjugated antibodies goat anti-mouse and goat anti-rabbit IgG from Bio-Rad Laboratories (Hercules, CA, USA); secondary Alexa Fluor-conjugated antibodies (anti-mouse and anti-rabbit) and DAPI from Jackson ImmunoResearch (West Grove, PA, USA).

### Cell isolation and culture

Primary human PSCs obtained from tumor tissue sampled from surgical resection specimens with PDAC, were isolated and cultured by the outgrowth method as previously described [29, 30]. Cultures were established and propagated from ten different patients (designated as PSC-1 -2, − 3, − 4, − 5, − 6, − 7, − 8, − 9, and − 10). None of the ten patients received neoadjuvant therapy. Clinicopathological features of tumors are provided in Additional file 1: Table S1. Assessment of morphology and expression analysis of αSMA and vimentin was performed to determine the purity of PSCs. PSC cultures between passage 3 and 8 were used for all experiments. The PSC cultures as well as the commercial PCC lines AsPC-1 (ATCC® CRL-1682™), BxPC-3 (ATCC® CRL-1687™), Capan-2 (ATCC® HTB-80™), HPAF-II (ATCC® CRL-1997™), MIA PaCa-2 (ATCC® CRL-1420™), PANC-1 (ATCC® CRL-1469™) and SW-1990 (ATCC® CRL-2172™) obtained from American Type Culture Collection (ATCC, Manassas, VA, USA) were cultured and maintained in DMEM supplemented with 10% FBS, 1% Pen-Strep and 1% amphotericin B and tested for mycoplasma contamination. None of the PCC lines are listed in the database of the International Cell Line Authentication Committee (ICLAC). For direct co-culture equal numbers of PSCs were seeded together with PCCs, while in indirect co-culture PCCs were incubated with PSC-conditioned medium (PSC-CM), as described in Additional file 2: Figure S1.

### Immunocytochemistry

Immunocytochemistry of PSCs was performed as described previously [31]. Briefly, PSCs cultured in 96-well plates were fixed in 4% formaldehyde, blocked in BSA and incubated overnight with primary antibodies against αSMA (1:50) and vimentin (1:200). The next day, cells were stained with Alexa Fluor-conjugated secondary antibodies (1:200) and DAPI was used for nuclear staining. Images were captured using EVOS FLoid Cell Imaging Station (Thermo Fisher Scientific).

### Preparation of conditioned medium

The PSC-CM samples were prepared as described previously [31]. Briefly, sub-confluent PSC cultures were washed thoroughly with PBS and incubated for 48 h with fresh serum-free DMEM (SFM), conditioned medium was collected, centrifuged and stored at - 20 °C until further use.

### Chemosensitivity

Both PCCs and PSCs were cultured in 96-well plates at a density of 3000 cells/well and treated for 48 h with gemcitabine at a final concentration range of 0.01–100 μM. The cell viability and IC_50_ values were determined using the MTT assay, in which the degree of formazan crystals formation is relative to the number of viable cells. IC_50_ values were determined by calculating the amount of gemcitabine required for inhibition of cell growth by 50% compared to untreated controls. Furthermore, PCCs were either cultured together with PSCs for 96 h (direct co-culture) or incubated with PSC-CM (indirect co-culture) for 24 h, prior to treatment with gemcitabine for 48 h at a final concentration of 10 μM. The response to gemcitabine was evaluated with the MTT assay. The PSC-CM induced resistance to gemcitabine in various PCC lines was calculated using the following formula:

$$ \mathrm{Drug}\ \mathrm{resistance}\ \left(\%\right)=\frac{\%\mathrm{viability}\ \mathrm{in}\ \mathrm{PSC}-\mathrm{CM}-\%\mathrm{viability}\ \mathrm{in}\ \mathrm{SFM}}{\%\mathrm{viability}\ \mathrm{in}\ \mathrm{SFM}}\mathrm{X}\ 100 $$

### Secretome analysis

Proteomics-based analysis of PSC-CM samples was performed as described previously with some modifications [31]. Briefly, the PSC-CM samples were concentrated down to 5% of the original volume using 10 kDa cut off Amicon Ultra centrifugal filter devices. Subsequently, the proteins were reduced, alkylated and digested overnight using trypsin (Promega). The next day, the peptides were desalted and concentrated before they were submitted to mass spectrometry (MS). Each peptide mixture was analyzed in three technical replicates by nEASY-LC coupled to QExactive Plus (ThermoElectron, Bremen, Germany) with EASY Spray PepMap®RSLC column (C18, 2 μm, 100 Å, 75 μm × 50 cm). Protein identification was performed using Proteome Discoverer 2.1 (Thermo Fisher Scientific) and Mascot 2.6 (MatrixScience, London, UK) search engine. The search criteria for Mascot searches were: trypsin digestion with two missed cleavage allowed, Carbamidomethyl (C) as fixed modification and Acetyl (N-term), Gln- > pyro-Glu (N-term Q), Oxidation (M) as dynamic modifications. The parent mass tolerance was 10 ppm and MS/MS tolerance 0.1 Da. The SwissProt database for human entries supplemented with known contaminants provided by MaxQuant was used for the database searches. All of the reported protein identifications were statistically significant (*p* < 0.05) in Mascot and filtered in Proteome Discoverer for at least medium confidence identifications. The list of identified proteins were subjected to Kyoto Encyclopedia of Genes and Genomes (KEGG) database for pathway analysis [32] and Gene Ontology (GO) analysis was conducted using the DAVID Bioinformatics Database [33, 34]. The workflow of the procedure is illustrated in Additional file 3: Figure S2A.

### ELISA

The amount of soluble FN present in the conditioned medium obtained from ten different PSC cultures was measured using human fibronectin ELISA kit, according to the manufacturer’s protocol.

### Western blot analysis

Total cell lysates were prepared using Laemmli buffer, and aliquots of protein were separated by electrophoresis (SDS-PAGE), as described previously [31]. The proteins were transferred to nitro-cellulose membranes, blocked in 5% non-fat dry milk solution and incubated overnight with the primary antibodies as indicated. The next day, blots were washed and incubated with HRP-conjugated secondary antibodies at room temperature for 1 h. The blots were visualized with LumiGLO® (KPL, Gaithersburg, MD, USA) and their densitometric analysis was performed using Labworks Software (UVP, Cambridge, UK).

### Gemcitabine uptake

PCCs cultured to confluence in 96-well plates were washed with PBS and incubated with 100 μL transport buffer (20 mM Tris, 3 mM K_2_HPO_4_, 5 mM glucose, 145 mM NaCl, 1 mM MgCl_2_, and 1.2 mM CaCl_2_) containing 50 nM [^3^H]-gemcitabine at 37 °C for 4 h. Subsequently, cells were lysed by incubation with 0.2 M NaOH for 15 min and lysates were then added to the scintillation tubes containing Ultima Gold. Cell-associated radioactivity in counts per minute (CPM) was determined using a liquid scintillation counter. Protein concentration of cell lysates was determined using BCA protein assay kit. [^3^H]-gemcitabine transport was calculated by normalizing CPM to protein concentration for each well.

### Statistical analysis

All values are expressed as a mean ± standard error of mean (SEM). Statistical analysis of the results was performed using GraphPad Prism 6 (GraphPad Software), by an unpaired two-tailed Student’s t test or by two-way ANOVA, followed by Tukey’s post hoc test, with a value of *p* < 0.05 considered statistically significant.

## Results

### Phenotypic characterization of the human primary PSC cultures

Expression of proteins considered characteristic of PSCs was analyzed by immunofluorescence and western blot analysis. All primary PSC cultures examined exhibited a fibroblast-like morphology that is characteristic of PSCs, and showed strong expression of the activation marker αSMA and the mesenchymal marker vimentin, consistent with activated PSCs (Additional file 4: Figure S3A, B). GFAP was not detected in any of the PSC cultures (*data not shown*), consistent with the reported loss of expression during culturing [35]. None of the PSC cultures showed expression of the epithelial marker EpCAM (*data not shown*), excluding the possibility of contamination with epithelial cells during isolation.

### Gemcitabine dose response chemosensitivity in PSCs and PCCs

**Fig. 1:**
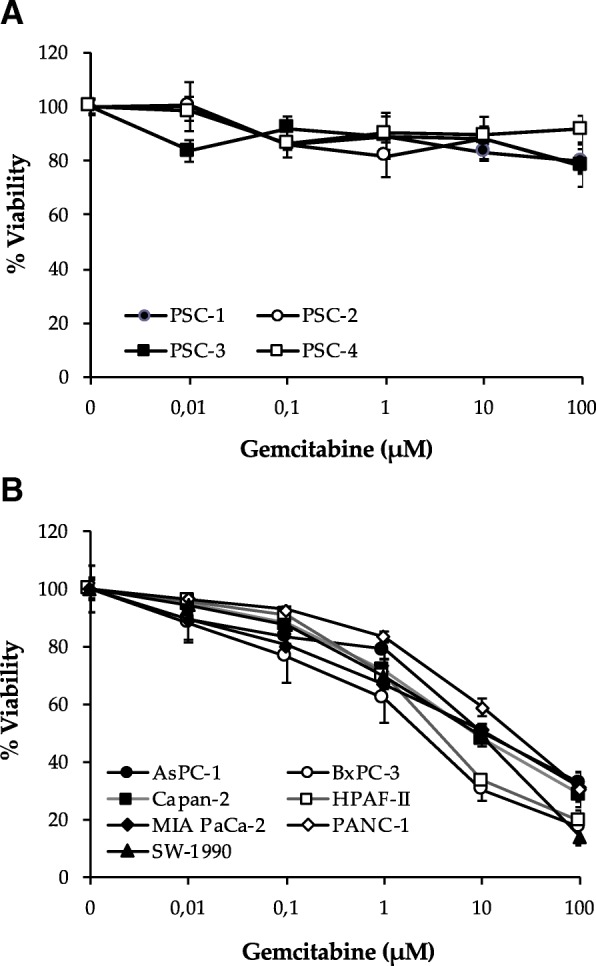
PSCs are resistant to gemcitabine-induced cytotoxicity while gemcitabine dose response effects differ among various PCC lines. PDAC-derived human PSCs (**a**) and PCCs (**b**) seeded on 96-well plates at a density of 3000 cells/well, were incubated with increasing concentrations of gemcitabine for 48 h and evaluated for cell viability using the MTT assay. Data are mean ± SEM of triplicate determinations. PCC, pancreatic cancer cell; PSC, pancreatic stellate cell

MTT-based evaluation of dose response to gemcitabine in terms of cell viability in four PSC cultures revealed that these cells were resistant to the cytotoxic actions of gemcitabine. At a single concentration of gemcitabine (10 μM) an average reduction of cell viability by merely 12% was observed (Fig. 1a). In contrast, gemcitabine differentially reduced the cell viability in the seven PCC lines studied (Fig. 1b). MTT assay revealed that gemcitabine dose dependently inhibited PCC viability and, at a single concentration of gemcitabine (10 μM), the decrease in cell viability was 50, 70, 52, 66, 49, 41 and 51% in AsPC-1, BxPC-3, Capan-2, HPAF-II, MIA PaCa-2, PANC-1 and SW-1990, respectively (Fig. 1b). Notably, a significant cell population in each PCC line was observed to be inherently resistant to gemcitabine, whereby the size of this subpopulation ranged from 14% of the total cell population in SW-1990 to 33% in AsPC-1 (Fig. 1b). The IC_50_ values suggested that PANC-1 (IC_50_ = ~ 20 μM) and BxPC-3 (~ 2 μM) were least and most sensitive to gemcitabine, respectively (Table 1), which is in accordance with previous data [36].

**Table 1:** IC_50_ values for gemcitabine in different PCC lines were calculated using GraphPad Prism software 6.0

Cell line	IC_50_ (μM)
PANC-1	20.5
AsPC-1	13.9
Capan-2	9.7
MIA PaCa-2	9.5
SW-1990	6.2
HPAF-II	4.3
BxPC-3	2.0

### Effect of PSCs and their secretome on gemcitabine chemosensitivity of PCCs

**Fig. 2:**
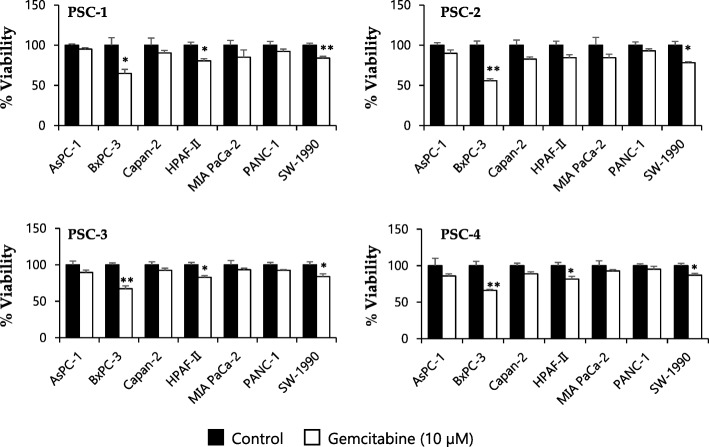
In direct co-culture with PSCs, PCCs develop resistance to gemcitabine. Equal numbers of PSCs and PCCs seeded together on 96-well plates, cultured for 4 days, were incubated with a single concentration of gemcitabine (10 μM) for 48 h and evaluated for cell viability using MTT assay. Data are mean ± SEM of triplicate determinations. *p < 0.05, **p < 0.01 for control vs gemcitabine. PCC, pancreatic cancer cell; PSC, pancreatic stellate cell

Each of the seven different PCCs were each co-cultured with four different PSC cultures for 96 h and evaluated for the response to gemcitabine at single concentration of 10 μM, using the MTT assay. Compared to the cell survival observed in PCCs alone (Fig. 1b), the cancer cells in co-cultures developed a considerable degree of resistance to gemcitabine, as indicated by a high cell survival than in monoculture (Fig. 2). For instance, co-culture with PSC-1 reduced cell viability by a mere 9, 35, 9, 19, 15, 7 and 16% for AsPC-1, BxPC-3, Capan-2, HPAF-II, MIA PaCa-2, PANC-1 and SW-1990, respectively. Similar responses were observed for co-cultures with three further PSC culture (Fig. 2).

**Fig. 3:**
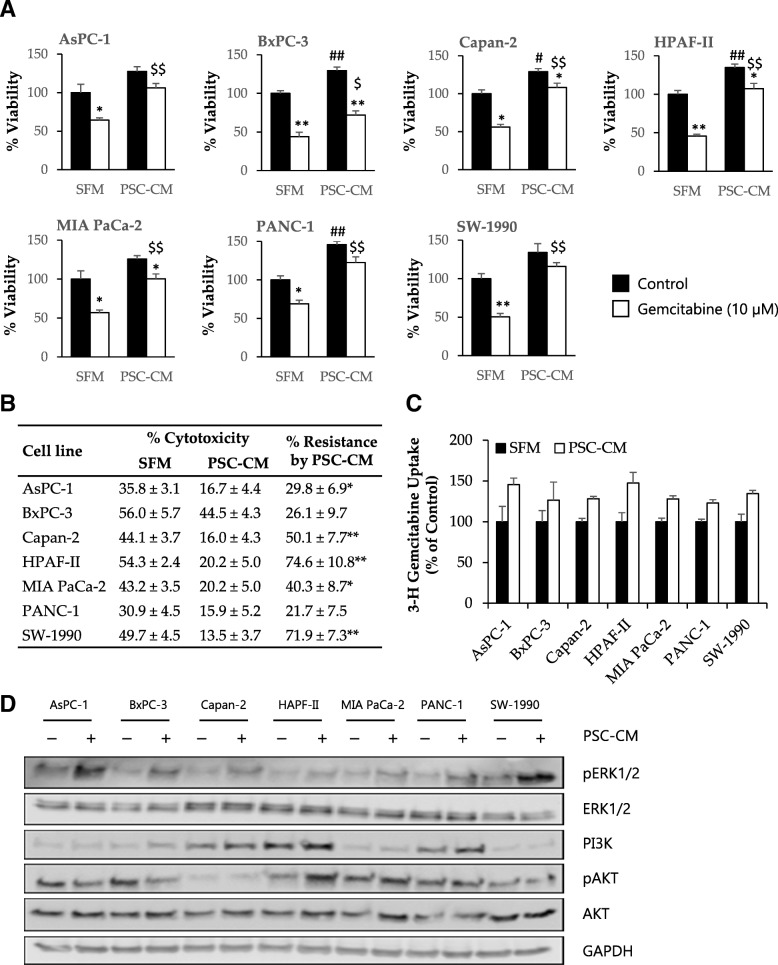
PSC-CM induces resistance to gemcitabine in PCCs but does not affect gemcitabine uptake. PCCs seeded on 96-well plates were incubated with SFM or PSC-CM for 24 h prior to incubation with (**a, b**) gemcitabine (10 μM) for 48 h or with (**c**) transport buffer containing [^3^H]-gemcitabine for 4 h. For (**a**), cell viability was determined using the MTT assay. *p < 0.05, **p < 0.01 for control vs gemcitabine; #*p* < 0.05, ##*p* < 0.01 and $*p* < 0.05, $$*p* < 0.01 for SFM vs PSC-CM in control and gemcitabine groups, respectively. **b** The table indicates gemcitabine-induced cytotoxicity in percentage, and PSC-CM-induced resistance to gemcitabine, calculated by relative reduction in cytotoxicity between SFM and PSC-CM. *p < 0.05, **p < 0.01 comparing SFM with PSC-CM. For **c**, gemcitabine uptake was determined by scintillation counting. Data are mean ± SEM of triplicate determinations. **d** The PCCs were lysed and proteins subjected to immunoblotting using anti-pERK1/2, anti-ERK1/2, anti-PI3K, anti-pAKT, and anti-AKT antibodies. GAPDH was used as a loading control. PCC, pancreatic cancer cell; PSC, pancreatic stellate cell; PSC-CM, PSC-conditioned medium; SFM, serum-free DMEM

To evaluate the effect of PSCs-secreted soluble factors on cancer cell chemosensitivity, PCCs were incubated with PSC-CM for 24 h and analyzed for the response to the cytotoxic effects of gemcitabine using the MTT assay (Fig. 3a). All PCC lines studied developed a certain degree of resistance to gemcitabine, with the highest resistance being observed in HPAF-II (~ 75%). Although PANC-1 and BxPC-3 cells displayed a modest resistance (20–25%), this change was not significant (Fig. 3a and b). Notably, across the cell lines that were studied, co-culture with PSC-CM had no significant effect on the uptake of radiolabeled gemcitabine in PCCs (Fig. 3c).

Of the many important signaling pathways that play a role in the pathobiology of PDAC, the ERK and PI3K/AKT signaling pathways are known to be also implicated in gemcitabine resistance [37, 38]. Substantiating the putative signaling effects of soluble factors in PSC-CM on the PCCs, a variable degree of increased phosphorylation of ERK1/2 was observed in all PCC lines upon incubation with PSC-CM (Fig. 3d), whereas activity of AKT or PI3K remained largely unaltered in the PCCs with the exception of HPAF-II, which displayed an increase in AKT phosphorylation (Fig. 3d).

### Secretome analysis of PSC-CM

**Fig. 4:**
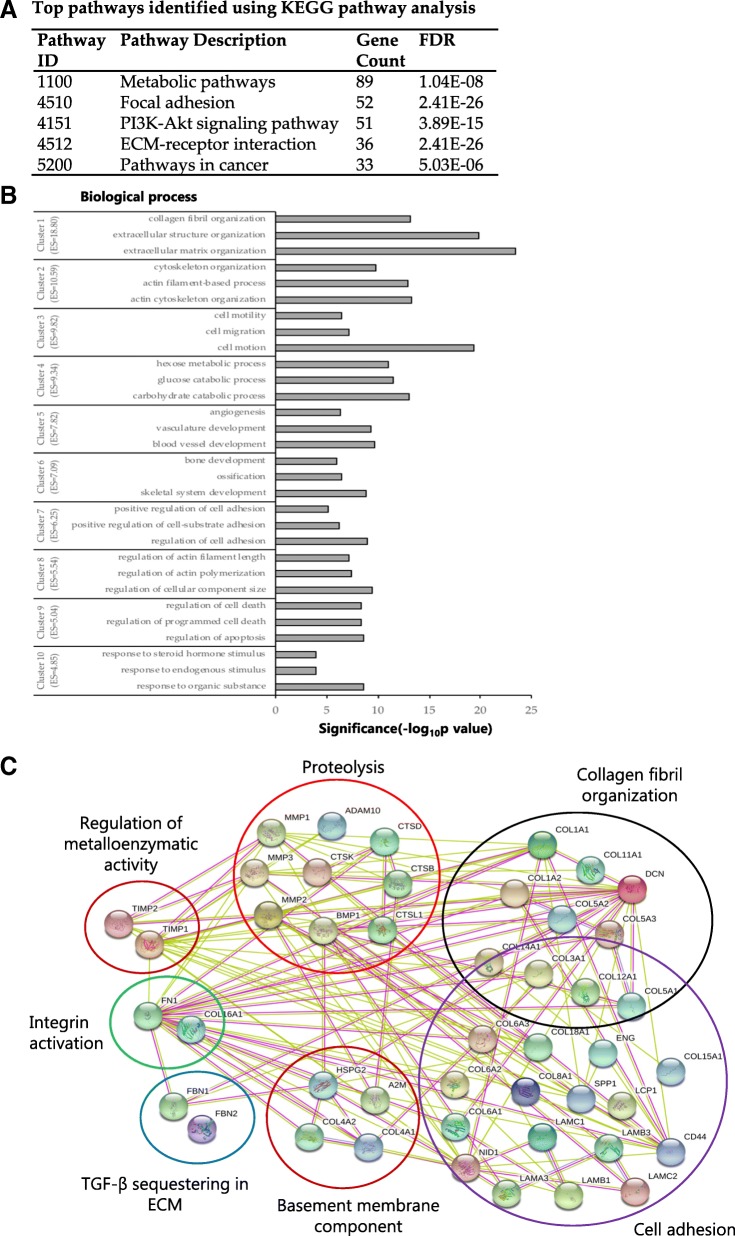
Proteome profiling of PSC-CM. Conditioned medium from ten different PSC cultures were subjected to proteomics analysis using LC-MS/MS. **a** Top five KEGG pathways identified. **b** The proteins detected by LC-MS/MS were interrogated in terms of functional annotation by DAVID Bioinformatics Resource tool. The representative GO terms cluster groups with top 10 enrichment score are presented. The horizontal axis represents the significance (*p* value) for each term, while the vertical axis represents the GO categories for biological processes. **c** STRING network map of proteins involved in ECM remodeling and their categories based on molecular function. ECM, extracellular matrix; ES, enrichment score; FDR, false discovery rate; GO, gene ontology; KEGG, kyoto encyclopedia of genes and genomes; PSC, pancreatic stellate cell; STRING search tool for the retrieval of interacting genes/proteins

The composition of conditioned medium from various PSC cultures was investigated by proteomics-based analysis of the secretome preparations. A total of 796 different proteins (*Homo sapiens*) were identified, after the protein grouping performed by MaxQuant software. A complete list of all proteins together with their identification parameters are provided in Additional file 5: Table S2. The proteins with a number of peptides with high-medium confidence identified was between 200 and 600, across the ten PSC cultures studied (Additional file 3: Figure S2B). The identified proteins mainly belonged to the structural cellular components such as exosomes, extracellular vesicles and membrane-bound vesicles. Furthermore, to facilitate the interpretation of the results, a functional interaction network of the identified proteins was searched using the STRING software. The top five enriched KEGG pathway categories of the identified proteins are listed in Fig. 4a. To determine the functional role of the PSC-secreted proteins, a GO analysis was undertaken using the DAVID bioinformatic resource tool, which revealed a significant representation of biological process categories that were mainly related to ECM structure and organization, cytoskeleton organization, cell motility and adhesion, metabolism, regulation of cell death, and response to stimuli (Fig. 4b). A GO analysis also revealed a significant representation of molecular processes categories that were mainly related to carbohydrate and polysaccharide binding, enzyme activity, and calcium biding (Additional file 3: Figure S2C). GO functional annotation chart for KEGG analysis is presented in Additional file 6: Table S3. Furthermore, a total of 47 of all the identified proteins that are involved in ECM remodeling (Table 2), were grouped based on molecular functions as indicated (Fig. 4c).

**Table 2:** List of ECM remodeling proteins identified in PSC secretome

Accession	Protein description	Gene symbol	Molecular weight [kDa]	Coverage
P02751	Fibronectin	FN1	262.5	66.7
P08123	Collagen alpha-2(I) chain	COL1A2	129.2	82.3
P02452	Collagen alpha-1(I) chain	COL1A1	138.9	81.1
P02461	Collagen alpha-1(III) chain	COL3A1	138.5	55.3
P12109	Collagen alpha-1(VI) chain	COL6A1	108.5	61.1
P35555	Fibrillin-1	FBN1	312.0	44.7
P12111	Collagen alpha-3(VI) chain	COL6A3	343.5	48.1
P08253	72 kDa type IV collagenase	MMP2	73.8	73.5
Q99715	Collagen alpha-1(XII) chain	COL12A1	332.9	35.0
P11047	Laminin subunit gamma-1	LAMC1	177.5	38.1
P98160	Heparan sulfate proteoglycan core protein	HSPG2	468.5	26.3
P08572	Collagen alpha-2(IV) chain	COL4A2	167.4	41.1
P03956	Interstitial collagenase	MMP1	54.0	65.9
P05997	Collagen alpha-2(V) chain	COL5A2	144.8	50.9
P01033	Metalloproteinase inhibitor 1	TIMP1	23.2	58.9
P20908	Collagen alpha-1(V) chain	COL5A1	183.4	21.0
P07585	Decorin	DCN	39.7	54.3
P12110	Collagen alpha-2(VI) chain	COL6A2	108.5	44.2
P16035	Metalloproteinase inhibitor 2	TIMP2	24.4	67.7
P07942	Laminin subunit beta-1	LAMB1	197.9	32.7
P02462	Collagen alpha-1(IV) chain	COL4A1	160.5	15.5
P14543	Nidogen-1	NID1	136.3	23.4
P12107	Collagen alpha-1(XI) chain	COL11A1	181.0	14.2
P01023	Alpha-2-macroglobulin	A2M	163.2	33.2
P07858	Cathepsin B	CTSB	37.8	46.3
P13497	Bone morphogenetic protein 1	BMP1	111.2	26.7
P16112	Aggrecan core protein	ACAN	250.0	10.2
P16070	CD44 antigen	CD44	81.5	6.3
Q13443	Disintegrin and metalloproteinase domain-containing protein 9	ADAM9	90.5	5.5
P13796	Plastin-2	LCP1	70.2	12.8
Q07092	Collagen alpha-1(XVI) chain	COL16A1	157.7	5.9
P27658	Collagen alpha-1(VIII) chain	COL8A1	73.3	18.1
P07339	Cathepsin D	CTSD	44.5	38.3
P08254	Stromelysin-1	MMP3	53.9	24.1
P35556	Fibrillin-2	FBN2	314.6	4.1
Q05707	Collagen alpha-1(XIV) chain	COL14A1	193.4	9.4
Q13751	Laminin subunit beta-3	LAMB3	129.5	8.7
Q13753	Laminin subunit gamma-2	LAMC2	130.9	8.5
O14672	Disintegrin and metalloproteinase domain-containing protein 10	ADAM10	84.1	15.1
P07711	Cathepsin L1	CTSL1	37.5	20.4
P10451	Osteopontin	SPP1	35.4	19.1
P17813	Endoglin	ENG	70.5	8.2
Q16787	Laminin subunit alpha-3	LAMA3	366.4	2.8
P39059	Collagen alpha-1(XV) chain	COL15A1	141.6	6.2
P39060	Collagen alpha-1(XVIII) chain	COL18A1	178.1	5.2
P25940	Collagen alpha-3(V) chain	COL5A3	172.0	2.7
P43235	Cathepsin K	CTSK	36.9	5.5

### Effect of PSC-secreted FN on PCC chemoresistance

**Fig. 5:**
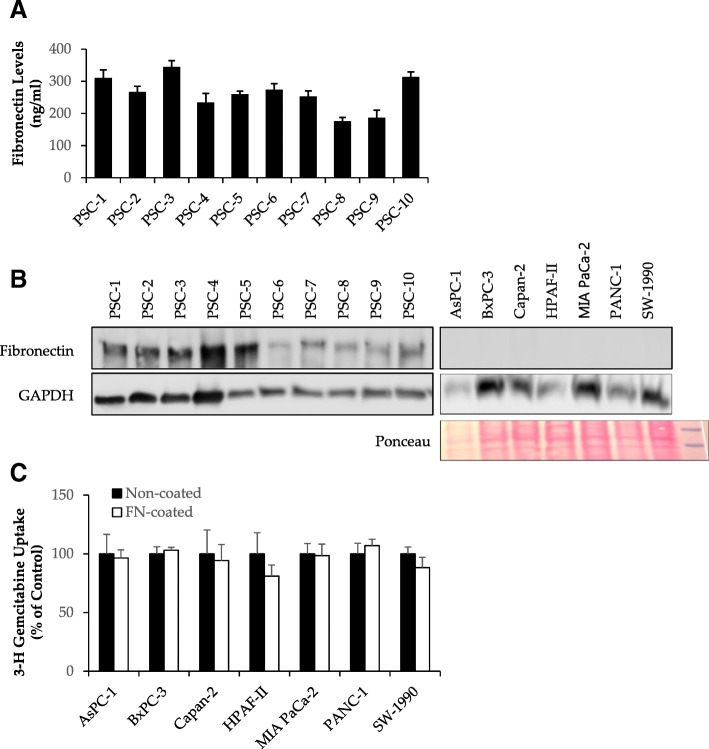
Measurement of FN content in PSC-CM and its effect on gemcitabine uptake by PCCs. **a** Amount of soluble FN in conditioned medium from ten different PSC cultures was measured by ELISA. **b** Both PCCs and PSCs were lysed and proteins subjected to immunoblotting using anti-FN antibody. GAPDH was used as a loading control. For PCCs, ponceau image also indicates loading. **c** Cancer cells seeded on 96-well plates with- or without FN-coating were incubated for 24 h prior to incubation with transport buffer containing [^3^H]-gemcitabine for 4 h. Data are mean ± SEM of triplicate determinations. FN, fibronectin; PCC, pancreatic cancer cell; PSC, pancreatic stellate cell

**Fig. 6:**
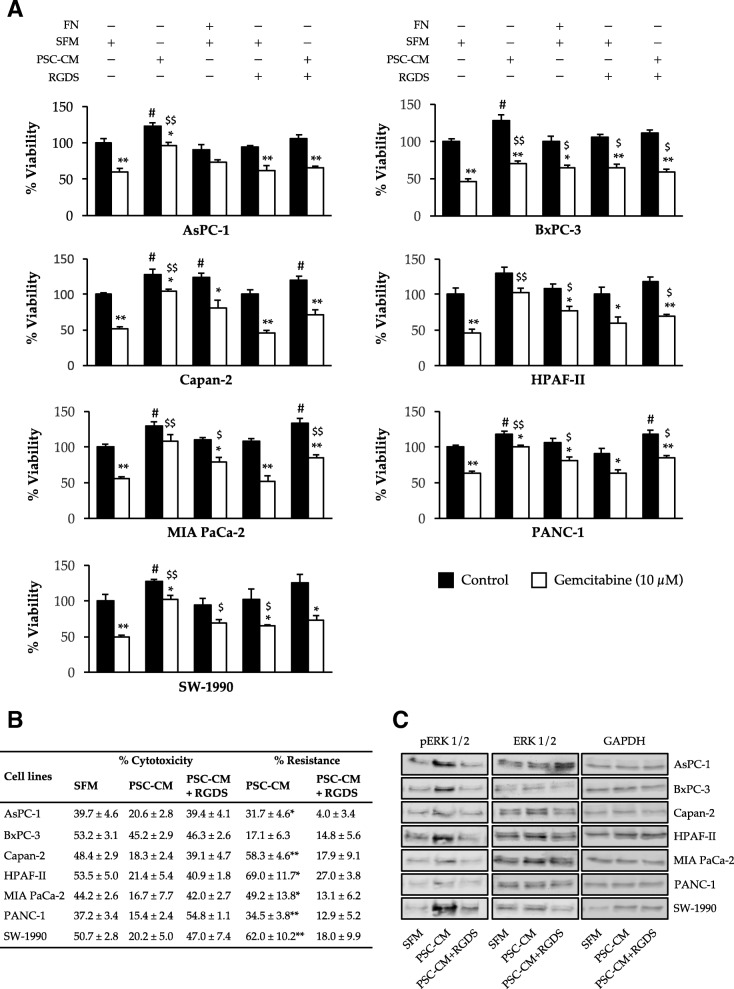
FN-inhibitor blocks chemoresistance inducing effect of PSC-secreted FN on PCCs via downregulation of ERK1/2 phosphorylation. **a** PCCs seeded on 96-well plates with- or without FN-coating as indicated. Cell were incubated with SFM or PSC-CM for 24 h and/or RGDS (FN inhibitor; 20 μM) for 4 h prior to incubation with gemcitabine (10 μM) for 48 h. Cell viability was determined using the MTT assay. *p < 0.05, **p < 0.01 for control vs gemcitabine; #*p* < 0.05, ##*p* < 0.01 and $*p* < 0.05, $$*p* < 0.01 for SFM vs PSC-CM/FN/RGDS in control (black bars) and gemcitabine (white bars), respectively. **b** The table indicates gemcitabine-induced cytotoxicity in percentage, PSC-CM-induced resistance to gemcitabine, and resistance following subsequent incubation with RGDS. The resistance developed was calculated by relative reduction in cytotoxicity between SFM and PSC-CM or PSC-CM + RGDS. *p < 0.05, **p < 0.01 comparing SFM with PSC-CM or SFM vs PSC-CM + RGDS. **c** The cells were lysed and proteins subjected to immunoblotting using anti-pERK1/2 and anti-ERK1/2 antibodies. GAPDH was used as a loading control. Data are the mean ± SEM of triplicate determinations. FN, fibronectin; PCC, pancreatic cancer cell; PSC, pancreatic stellate cell; PSC-CM, PSC-conditioned medium; SFM, serum-free DMEM

In the PSC secretome, FN was detected with one of the highest number of peptides. The levels of soluble FN in conditioned medium from the ten PSCs cultures were in the range 175–350 ng/ml (Fig. 5a). Western blot analysis revealed that only the PSCs expressed FN, whereas all the PCC lines studied were negative (Fig. 5b), which is in accordance with previous findings [39]. Gemcitabine uptake capacity was not affected by FN-coating in any of the PCC lines studied (Fig. 5c). To evaluate the effect of FN on chemoresistance development, PCCs were cultured on FN-coated plates, and all cell lines with the exception of BxPC-3, displayed partial loss of the chemosensitivity to gemcitabine (Fig. 6a and b). However, the observed effect was considerably weaker than that of co-culture with PSC-CM (Fig. 3). To evaluate the effect of collagen - another major PSC-CM component – on chemoresistance development, PCCs were cultured on collagen-coated plates, which showed that none of the cell lines displayed reduction or loss of chemosensitivity to gemcitabine (Additional file 7: Figure S4). Furthermore, PCCs pre-incubated with PSC-CM and further incubated with RGDS, a FN inhibitor (H-Arg-Gly-Asp-Ser-OH), regained sensitivity to gemcitabine to some extent (Fig. 6a and b). Interestingly, in all PCC lines, treatment with RGDS also reversed the increase in phosphorylation of ERK1/2 to basal levels that had occurred during co-culture with PSC-CM (Fig. 6c). These findings were further confirmed by treatment with PD98059, a MEK/ERK inhibitor, which resulted in regained sensitivity to gemcitabine to an extent similar to that of RGDS (Additional file 8: Figure S5).

## Discussion

Crosstalk between mesenchymal stromal cells and epithelial cancer cells has recently been implicated in the development of chemoresistance in pancreatic cancer. In particular, increasing evidence suggest that PSCs in the tumor stroma are both direct and indirect drivers of pancreatic cancer chemoresistance [23]. Since the 1997 report by Burris et al. [8], gemcitabine has remained a cornerstone of pancreatic cancer chemotherapy despite only marginal effects on patient survival, to a large extent caused by the high intrinsic (de novo) and acquired (therapy-induced) chemoresistance [40]. Thus, knowledge about the underlying mechanisms of drug resistance in PDAC is critical in attempts to develop new and effective treatment regimens for this lethal condition.

In the present study we performed a proteomics-based analysis of the PSC secretome to identify PSC-secreted factors that possibly affect PCCs in signaling pathways involved in the development of resistance to gemcitabine. The analysis resulted in identification of 796 proteins, the majority of which were ECM related proteins including FN and collagens. When cultured on FN-coated surface, all PCC lines tested displayed to a varying degree an increased resistance to gemcitabine, suggesting that ECM proteins are implicated in resistance-induction, in accordance with a previous report by Miyamoto et al. [28]. We observed that both in indirect and direct co-culture, PSCs induced a varying degree of gemcitabine resistance in all PCC lines tested. Notably, a synthetic FN inhibitor (RGDS peptide, which blocks integrin receptor function) counteracted to a limited extent resistance-induction by the PSCs, suggesting that PSC-secreted FN plays a key role in the development of gemcitabine resistance. However, the effect of PSC-CM on the development of chemoresistance was significantly higher compared to the effect of FN alone, which suggests that secretome components other than FN may also contribute to chemoresistance to gemcitabine.

Several tumor-modifying interactions between PSCs and PCCs have been reported in the literature, including effects of PSCs on cancer cell proliferation, migration and invasion as well as resistance to chemotherapeutic agents [10, 35, 41–44]. The panel of PCCs examined in the present study differ in their sensitivity to gemcitabine, as the IC_50_ for gemcitabine was approximately ten times higher in the relatively resistant PANC-1 cells compared to the most sensitive BxPC-3 cells. Upon direct co-culture with the tumor-derived PSCs, all the PCC lines examined became relatively resistant, albeit to a variable degree, indicating possible cell-line specific effects. In this context it should be recognized that the cancer cells differ in their mutation status; notably the BxPC-3 cell line is the only one with wild type KRAS [45]. Although several mechanisms could conceivably account for the observed loss of sensitivity to gemcitabine during direct co-culture, effects of PSC-secreted components on cancer cells seems an obvious possibility. This was substantiated with the findings from indirect co-culture experiments in which incubation of PCCs with PSC-CM resulted in the development of varying degrees of resistance to gemcitabine across all cell lines examined. Notably, direct co-culture experiments also suggest that, despite the differences in origin (tumor biopsies from different patients), PSCs modulated the chemosensitivity to gemcitabine of neighboring cancer cells to a similar extent.

Shortcomings of PDAC chemotherapy have been ascribed to poor drug availability as well as stromal factors with the ability to induce chemoresistance. Intracellular uptake of gemcitabine requires nucleoside transporters hENTs and hCNTs, and their expression correlate with gemcitabine sensitivity and overall survival among gemcitabine-treated PDAC patients. Loss of nucleoside transporters leads to development of resistance to gemcitabine [10, 39, 46, 47]. In the present study, neither PSC-CM nor FN had any significant effect on the overall gemcitabine uptake, suggesting that the observed loss of chemosensitivity may not be an effect of PSCs on the gemcitabine uptake capacity of PCCs. Furthermore, KEGG pathways analysis of the PSC secretome indicated that PSC secretion of FN could be a major determinant for induction of gemcitabine chemoresistance. Indeed, FN is a major ECM component that is primarily secreted by fibroblasts and cancer-associated fibroblasts [48, 49], and most of the biological effects of FN in as diverse processes as embryogenesis, wound healing and tissue homeostasis [48, 50–53] are mediated through integrins. The latter are a family of heterodimeric and transmembrane receptors that lead to activation of several signal transduction pathways, including ERK, p38 and PI3K/AKT [54, 55].

Both the extracellular signal-regulated kinase (ERK) pathway and the PI3K/AKT pathway are known to be important in the pathobiology of PDAC [56, 57] and also implicated in gemcitabine chemoresistance [37, 38]. High ERK1/2 activity has also been reported to contribute to the gemcitabine resistance in pancreatic cancer, by protecting tumor cells from chemotherapy-induced apoptosis [58, 59]. In this study, incubation of PCCs with PSC-CM-induced an increase in phosphorylation of ERK1/2, and this effect was counteracted by the synthetic FN inhibitor, suggesting that of many possible signaling pathways, PSCs mediate the resistance to gemcitabine via ERK1/2 activity. This was further confirmed by treatment with PD98059, a MEK/ERK inhibitor, which resulted in regained chemosensitivity for gemcitabine in PCCs. Notably, FN displayed profound inhibitory effects on ERK signaling whereas the gemcitabine sensitivity was not inhibited to the same extent, suggesting that other mechanisms or signaling pathways also might be involved. The PI3K/AKT pathway has been reported to be linked to anti-apoptotic signal transduction and chemoresistance in the PCC lines PK1 and PK8 as well as in an orthotopic xenograft model from the same cells [60, 61]. However, it was later reported by Arlt et al. [62], that no such role of PI3K/AKT was found in a study employing BxPc-3, Capan-1 and PancTu-1 cells. In the present study, no change in activity of AKT or PI3K was observed in response to PSC-CM across the seven different PCCs examined. A previous study by Sawai et al. [63] showed that the basement membrane proteins FN and collagen IV induced invasion of pancreatic cancer cells via increased ERK1/2 phosphorylation and that this effect was inhibited by RGDS peptide and β1-integrin antibody as well as by the MEK/ERK inhibitor PD98059. Thus, the results of the present study are consistent with the notion that fibronectin-integrin mediated ERK signaling is involved in pancreatic cancer cell proliferation and survival.

In addition to FN, other matrix components, particularly collagens, may contribute to stroma-induced chemoresistance [64]. High levels of expression of collagen genes were associated with drug resistance in ovarian and breast cancer cell lines [65, 66]. Knowledge regarding the role of different collagens in chemoresistance in pancreatic cancer is very limited. PANC-1 cells cultured in 3D collagen I have been reported to show increased gemcitabine resistance due to increased histone acetylation, also possibly affecting gene expression through activation of PI3K/AKT and ERK signaling pathways, leading to increased proliferation despite drug treatment [27, 67].

Despite recent advances in combination therapies, gemcitabine remains a cornerstone of neo-adjuvant, adjuvant, and palliative therapy for advanced PDAC [68]. A better understanding of the causes of gemcitabine chemoresistance, including the role of the tumor stroma, is critical to the development of novel treatment strategies. It seems obvious that developing new therapies for PDAC is a much more complex challenge than merely targeting the cancer cells alone. Recent studies in genetically modified mouse models and clinical trials targeting PSCs have shown conflicting results, with both positive and negative effects on cancer progression and responses to gemcitabine [69]. Understanding the aspects of PSC biology that promote therapy resistance and identifying the relevant signaling mechanisms are important steps in the development of effective stroma-targeting treatment strategies.

## Conclusions

**Fig. 7:**
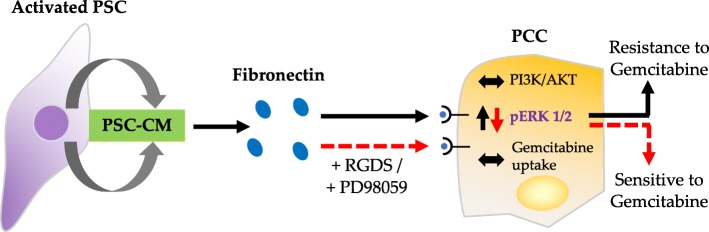
PSC-secreted fibronectin promotes chemoresistance to gemcitabine in PCCs. PSC-secreted fibronectin induces resistance to gemcitabine in PCCs via upregulation of ERK1/2 phosphorylation, while no change in gemcitabine uptake. Addition of RGDS (fibronectin receptor blocking agent) or PD98059 (MEK/ERK inhibitor) to PCCs incubated with PSC-CM protect from chemoresistance progression, via inhibition of ERK1/2 phosphorylation. PCC, pancreatic cancer cell; PSC, pancreatic stellate cell; PSC-CM, PSC-conditioned medium

In conclusion, the findings of the present study suggest that PSC-secreted FN promotes high ERK1/2 activity in cancer cells and thereby protects the PCCs from gemcitabine-induced cytotoxicity (Fig. 7). Combined use of an FN inhibitor and gemcitabine might result in reduced chemoresistance to gemcitabine. Further studies are needed to elucidate the mechanisms by which FN blocking agents added to gemcitabine-based chemotherapy might counteract chemoresistance in PDAC thereby providing better clinical outcomes.

## Additional files

Additional file 1: **Table S1.** Clinicopathological features of pancreatic adenocarcinoma (PADC) tumors. (DOCX 14 kb)

Additional file 2: **Figure S1.** Experimental set-up for co-culture experiments. Monolayer of PCCs (A), PSCs (B), direct co-culture of PCCs with PSCs (C) and indirect co-culture of PSCs with PCCs (D). PCC, pancreatic cancer cell; PSC, pancreatic stellate cell. (PPTX 61 kb)

Additional file 3: **Figure S2.** Secretome analysis of PSC-conditioned medium. (A) Flow-chart describing procedure for conditioned medium collection, processing and proteome analysis of the PSC secretome. (B) Conditioned medium collected from ten different PSC cultures were subjected to proteomic analysis by LC-MS/MS. Graph indicates the number of proteins identified with high confidence from each sample. (C) The proteins detected by LC-MS/MS were interrogated in terms of functional annotation by DAVID Bioinformatics Resource tool. The representative GO terms cluster groups with top 10 enrichment score are presented. The horizontal axis represents the significance (*p* value) for each term, while the vertical axis represents the GO categories for biological processes. GO, gene ontology; KEGG, kyoto encyclopedia of genes and genomes; PSC, pancreatic stellate cell; PSC-CM, PSC-conditioned medium; SFM, serum-free DMEM; STRING, search tool for the retrieval of interacting genes/proteins. (PPTX 3662 kb)

Additional file 4: **Figure S3.** Characterization of human PSCs. (A) PSCs immunostained with anti-αSMA (green) and anti-vimentin (red) antibodies. Nuclei stained with DAPI (blue). Scale bar = 100 μM. (B) The cells were lysed and proteins subjected to immunoblotting using anti-αSMA and anti-vimentin antibodies. GAPDH was used as a loading control. PSC, pancreatic stellate cell; αSMA, α-smooth muscle actin. (PPTX 4411 kb)

Additional file 5: **Table S2.** A complete list of all PSC secretome proteins together with their identification parameters. (XLSX 154 kb)

Additional file 6: **Table S3. ** Gene ontology functional annotation. (XLSX 15 kb)

Additional file 7: **Figure S4.** Effect of collagen on gemcitabine sensitivity. PCCs seeded on 96-well plates with- or without collagen-coating as indicated. Cells were incubated with SFM for 24 h prior to incubation with gemcitabine (10 μM) for 48 h. Cell viability was determined using the MTT assay. Data are the mean ± SEM of triplicate determinations. *p < 0.05, **p < 0.01 for control vs gemcitabine; #*p* < 0.05, for SFM vs collagen in control and gemcitabine groups. SFM, serum-free DMEM. (PDF 41 kb)

Additional file 8: **Figure S5.** Both FN-inhibitor (RGDS) and ERK-inhibitor (PD98059) block PSC-CM induced chemoresistance to gemcitabine. PCCs seeded on 96-well plates were incubated with SFM or PSC-CM for 24 h and/or RGDS (20 μM) or PD98059 (20 μM) for 4 h prior to incubation with gemcitabine (10 μM) for 48 h. Cell viability was determined using the MTT assay. Data are the mean ± SEM of triplicate determinations. *p < 0.05, **p < 0.01 for control vs gemcitabine; #*p* < 0.05, ##*p* < 0.01 and $*p* < 0.05, $$*p* < 0.01 for SFM vs PSC-CM/RGDS/ PD98059 in control and gemcitabine, respectively. FN, fibronectin; PSC, pancreatic stellate cell; PSC-CM, PSC-conditioned medium; SFM, serum-free DMEM. (PDF 48 kb)

## Data Availability

The datasets used and/or analyzed during the current study are available from the corresponding author on reasonable requests.

## Abbreviations

CAFs: Cancer-associated fibroblasts
ECM: Extracellular matrix
FN: Fibronectin
PCC: Pancreatic cancer cell
PDAC: Pancreatic ductal adenocarcinoma
PSC: Pancreatic stellate cell
PSC-CM: PSC-conditioned medium
SFM: Serum-free DMEM
