# Supplementary material for: Secretion of fibronectin by human pancreatic stellate cells promotes chemoresistance to gemcitabine in pancreatic cancer cells
Source: BMC Cancer. 2019 Jun 17;19:596. doi: 10.1186/s12885-019-5803-1 (PMC6580453; doi:10.1186/s12885-019-5803-1)
Supplement: Supplementary file 1 — Table S1. Clinicopathological features of pancreatic adenocarcinoma (PADC) tumors. (DOCX 14 kb) [file 12885_2019_5803_MOESM1_ESM.docx]

**Additional File 1 _Table S1**. Clinicopathological features of pancreatic adenocarcinoma (PADC) tumors

| **PSC ID** | **TN-stage** | **Tumour size (mm)** | **Tumour site** | **Grade** | **L** | **V** | **Pn** | **R** |
| --- | --- | --- | --- | --- | --- | --- | --- | --- |
| PSC-1 | T3N1 | 26 | head | 3 | 1 | 1 | 1 | 0 |
| PSC-2 | T3N1 | 35 | body | 2 | 1 | 1 | 1 | 1 |
| PSC-3 | T3N1 | 36 | head | 2 | 1 | 1 | 1 | 1 |
| PSC-4 | T3N1 | 40 | head | 2 | 1 | 1 | 1 | 1 |
| PSC-5 | T3N1 | 35 | head | 3 | 1 | 1 | 1 | 0 |
| PSC-6 | T3N1 | 30 | head | 2 | 1 | 1 | 1 | 0 |
| PSC-7 | T3N1 | 36 | head | 2 | 1 | 1 | 1 | 1 |
| PSC-8 | T3N1 | 32 | head | 2 to 3 | 1 | 1 | 1 | 1 |
| PSC-9 | T3N0 | 42 | head | 3 | 0 | 1 | 1 | 0 |
| PSC-10 | T3N1 | 38 | head | 2 to 3 | 1 | 1 | 1 | 1 |

PSC, pancreatic stellate cell. w
