# Supplementary material for: Secretion of fibronectin by human pancreatic stellate cells promotes chemoresistance to gemcitabine in pancreatic cancer cells
Source: BMC Cancer. 2019 Jun 17;19:596. doi: 10.1186/s12885-019-5803-1 (PMC6580453; doi:10.1186/s12885-019-5803-1)
Supplement: Supplementary file 2 — Figure S1. Experimental set-up for co-culture experiments. Monolayer of PCCs (A), PSCs (B), direct co-culture of PCCs with PSCs (C) and indirect co-culture of PSCs with PCCs (D). PCC, pancreatic cancer cell; PSC, pancreatic stellate cell. (PPTX 61 kb) [file 12885_2019_5803_MOESM2_ESM.pptx]

## Slide 1
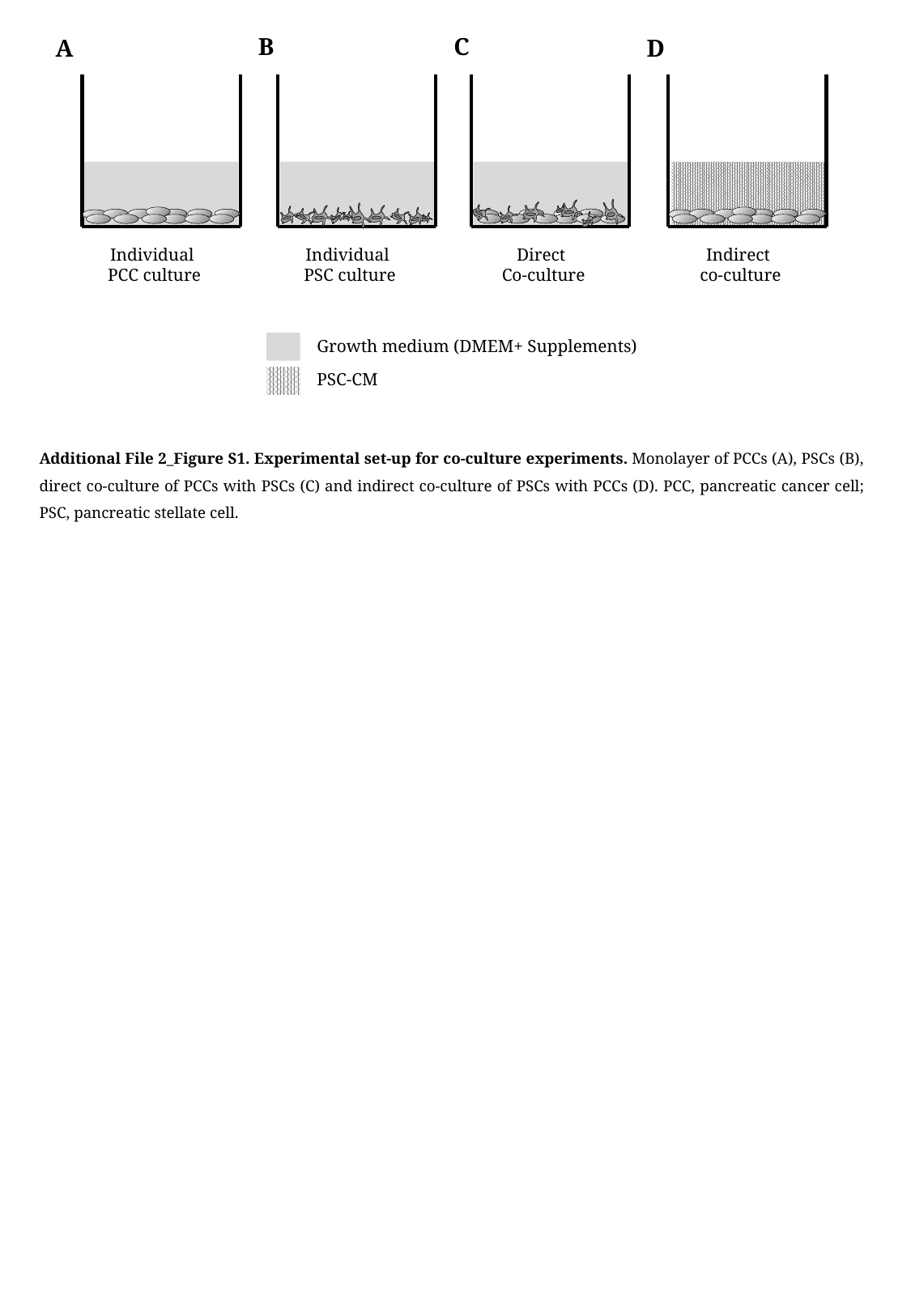

B
C
A
D
Individual
PCC culture
Individual
PSC culture
Direct
Co-culture
Indirect
co-culture
Growth medium (DMEM+ Supplements)
PSC-CM
Additional File 2_Figure S1. Experimental set-up for co-culture experiments. Monolayer of PCCs (A), PSCs (B), direct co-culture of PCCs with PSCs (C) and indirect co-culture of PSCs with PCCs (D). PCC, pancreatic cancer cell; PSC, pancreatic stellate cell.
