# Supplementary material for: Secretion of fibronectin by human pancreatic stellate cells promotes chemoresistance to gemcitabine in pancreatic cancer cells
Source: BMC Cancer. 2019 Jun 17;19:596. doi: 10.1186/s12885-019-5803-1 (PMC6580453; doi:10.1186/s12885-019-5803-1)
Supplement: Supplementary file 3 — Figure S2. Secretome analysis of PSC-conditioned medium. (A) Flow-chart describing procedure for conditioned medium collection, processing and proteome analysis of the PSC secretome. (B) Conditioned medium collected from ten different PSC cultures were subjected to proteomic analysis by LC-MS/MS. Graph indicates the number of proteins identified with high confidence from each sample. (C) The proteins detected by LC-MS/MS were interrogated in terms of functional annotation by DAVID Bioinformatics Resource tool. The representative GO terms cluster groups with top 10 enrichment score are presented. The horizontal axis represents the significance (p value) for each term, while the vertical axis represents the GO categories for biological processes. GO, gene ontology; KEGG, kyoto encyclopedia of genes and genomes; PSC, pancreatic stellate cell; PSC-CM, PSC-conditioned medium; SFM, serum-free DMEM; STRING, search tool for the retrieval of interacting genes/proteins. (PPTX 3662 kb) [file 12885_2019_5803_MOESM3_ESM.pptx]

## Slide 1
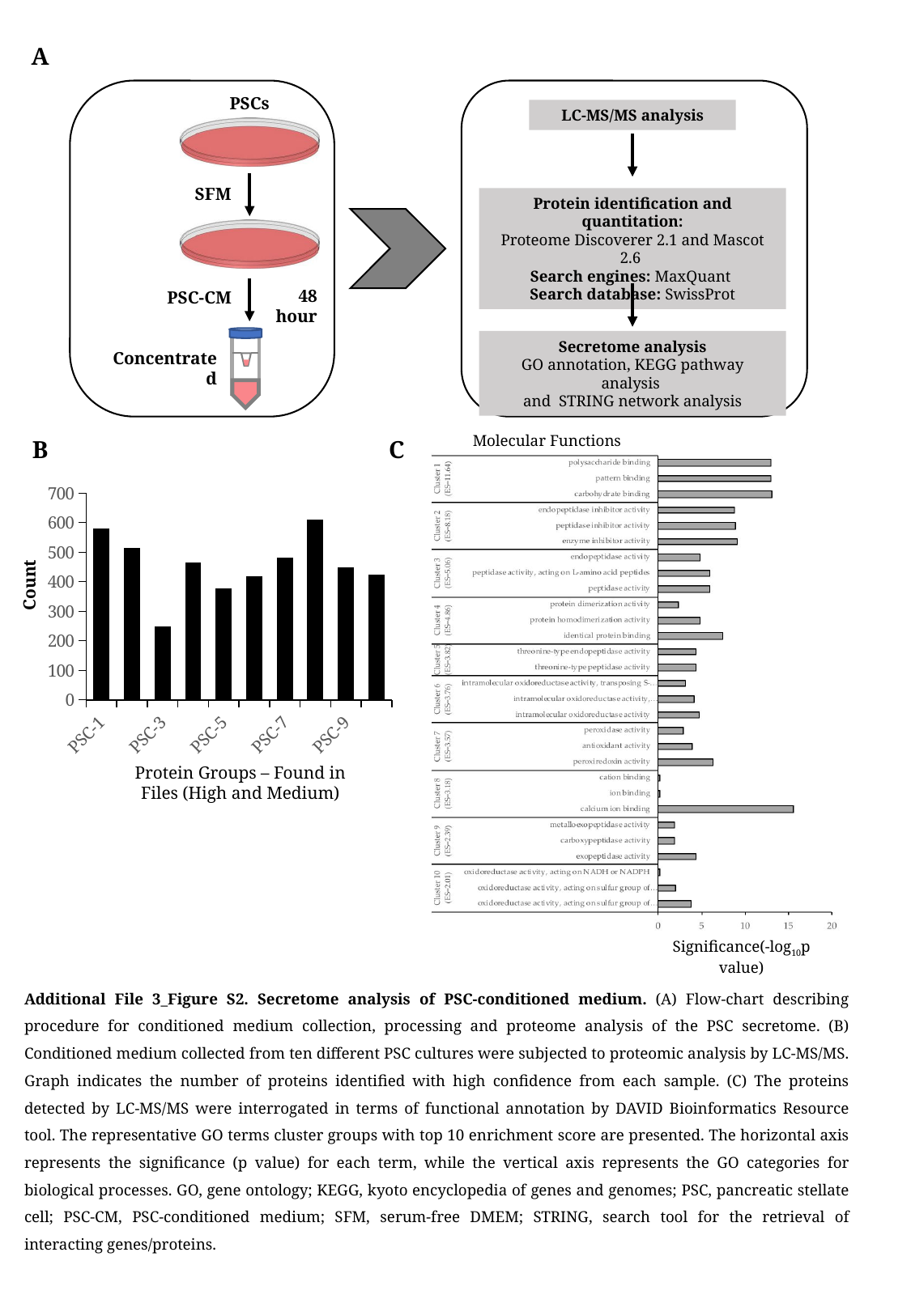

A
PSCs
SFM
48 hour
PSC-CM
Concentrated
LC-MS/MS analysis
Protein identification and quantitation:
Proteome Discoverer 2.1 and Mascot 2.6
Search engines: MaxQuant
Search database: SwissProt
Secretome analysis
GO annotation, KEGG pathway analysis
and STRING network analysis
B
C
Molecular Functions
### Chart
| Category | |
|---|---|
| PSC-1 | 580.0 |
| PSC-2 | 512.0 |
| PSC-3 | 247.0 |
| PSC-4 | 465.0 |
| PSC-5 | 376.0 |
| PSC-6 | 417.0 |
| PSC-7 | 480.0 |
| PSC-8 | 609.0 |
| PSC-9 | 447.0 |
| PSC-10 | 424.0 |Count
Protein Groups – Found in Files (High and Medium)
Significance(-log10p value)
Additional File 3_Figure S2. Secretome analysis of PSC-conditioned medium. (A) Flow-chart describing procedure for conditioned medium collection, processing and proteome analysis of the PSC secretome. (B) Conditioned medium collected from ten different PSC cultures were subjected to proteomic analysis by LC-MS/MS. Graph indicates the number of proteins identified with high confidence from each sample. (C) The proteins detected by LC-MS/MS were interrogated in terms of functional annotation by DAVID Bioinformatics Resource tool. The representative GO terms cluster groups with top 10 enrichment score are presented. The horizontal axis represents the significance (p value) for each term, while the vertical axis represents the GO categories for biological processes. GO, gene ontology; KEGG, kyoto encyclopedia of genes and genomes; PSC, pancreatic stellate cell; PSC-CM, PSC-conditioned medium; SFM, serum-free DMEM; STRING, search tool for the retrieval of interacting genes/proteins.
