# Supplementary material for: Secretion of fibronectin by human pancreatic stellate cells promotes chemoresistance to gemcitabine in pancreatic cancer cells
Source: BMC Cancer. 2019 Jun 17;19:596. doi: 10.1186/s12885-019-5803-1 (PMC6580453; doi:10.1186/s12885-019-5803-1)
Supplement: Supplementary file 4 — Figure S3. Characterization of human PSCs. (A) PSCs immunostained with anti-αSMA (green) and anti-vimentin (red) antibodies. Nuclei stained with DAPI (blue). Scale bar = 100 μM. (B) The cells were lysed and proteins subjected to immunoblotting using anti-αSMA and anti-vimentin antibodies. GAPDH was used as a loading control. PSC, pancreatic stellate cell; αSMA, α-smooth muscle actin. (PPTX 4411 kb) [file 12885_2019_5803_MOESM4_ESM.pptx]

## Slide 1
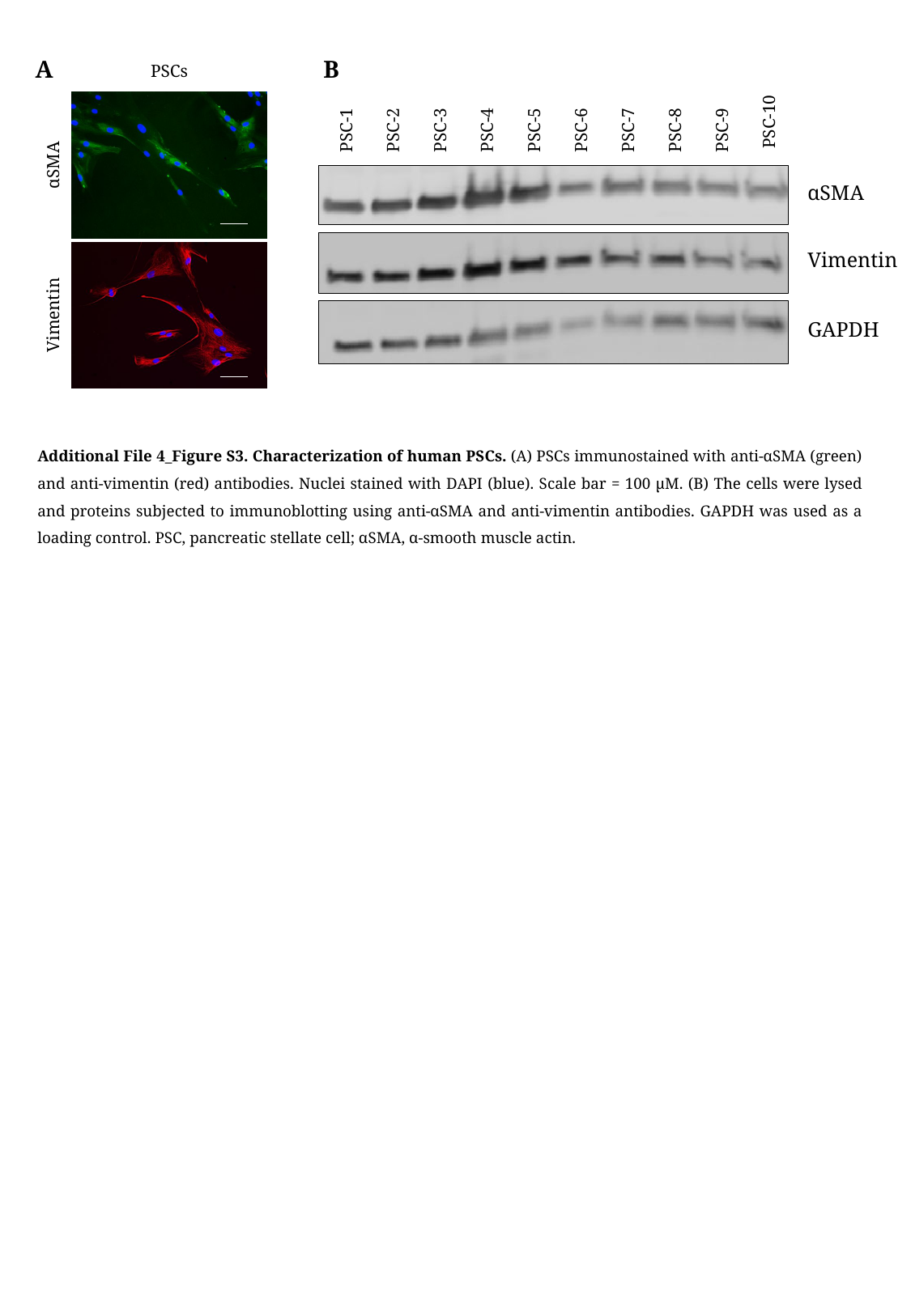

A
B
PSCs
ɑSMA
Vimentin
PSC-10
PSC-1
PSC-2
PSC-3
PSC-4
PSC-5
PSC-6
PSC-7
PSC-8
PSC-9
ɑSMA
Vimentin
GAPDH
Additional File 4_Figure S3. Characterization of human PSCs. (A) PSCs immunostained with anti-αSMA (green) and anti-vimentin (red) antibodies. Nuclei stained with DAPI (blue). Scale bar = 100 µM. (B) The cells were lysed and proteins subjected to immunoblotting using anti-αSMA and anti-vimentin antibodies. GAPDH was used as a loading control. PSC, pancreatic stellate cell; αSMA, α-smooth muscle actin.
