# Supplementary material for: Secretion of fibronectin by human pancreatic stellate cells promotes chemoresistance to gemcitabine in pancreatic cancer cells
Source: BMC Cancer. 2019 Jun 17;19:596. doi: 10.1186/s12885-019-5803-1 (PMC6580453; doi:10.1186/s12885-019-5803-1)
Supplement: Supplementary file 7 — Figure S4. Effect of collagen on gemcitabine sensitivity. PCCs seeded on 96-well plates with- or without collagen-coating as indicated. Cells were incubated with SFM for 24 h prior to incubation with gemcitabine (10 μM) for 48 h. Cell viability was determined using the MTT assay. Data are the mean ± SEM of triplicate determinations. *p < 0.05, **p < 0.01 for control vs gemcitabine; #p < 0.05, for SFM vs collagen in control and gemcitabine groups. SFM, serum-free DMEM. (PDF 41 kb) [file 12885_2019_5803_MOESM7_ESM.pdf]

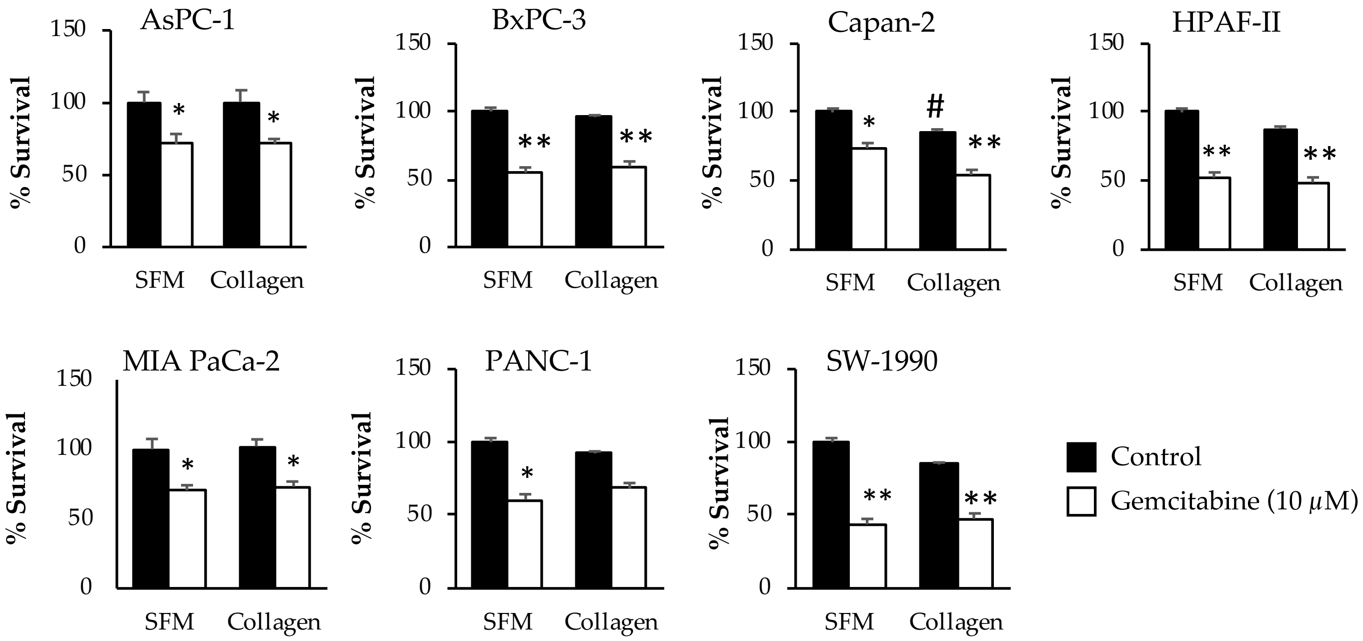

Additional File 7_Figure S4. Effect of collagen on gemcitabine sensitivity. PCCs seeded on 96-well plates with- or without collagen-coating as indicated. Cells were incubated with SFM for 24 hours prior to incubation with gemcitabine (10 μM) for 48 hours. Cell viability was determined using the MTT assay. Data are the mean ± SEM of triplicate determinations. *p<0.05, **p<0.01 for control vs gemcitabine; #p<0.05, for SFM vs collagen in control and gemcitabine groups. SFM, serum-free DMEM.
