# Supplementary material for: Secretion of fibronectin by human pancreatic stellate cells promotes chemoresistance to gemcitabine in pancreatic cancer cells
Source: BMC Cancer. 2019 Jun 17;19:596. doi: 10.1186/s12885-019-5803-1 (PMC6580453; doi:10.1186/s12885-019-5803-1)
Supplement: Supplementary file 8 — Figure S5. Both FN-inhibitor (RGDS) and ERK-inhibitor (PD98059) block PSC-CM induced chemoresistance to gemcitabine. PCCs seeded on 96-well plates were incubated with SFM or PSC-CM for 24 h and/or RGDS (20 μM) or PD98059 (20 μM) for 4 h prior to incubation with gemcitabine (10 μM) for 48 h. Cell viability was determined using the MTT assay. Data are the mean ± SEM of triplicate determinations. *p < 0.05, **p < 0.01 for control vs gemcitabine; #p < 0.05, ##p < 0.01 and $p < 0.05, $$p < 0.01 for SFM vs PSC-CM/RGDS/ PD98059 in control and gemcitabine, respectively. FN, fibronectin; PSC, pancreatic stellate cell; PSC-CM, PSC-conditioned medium; SFM, serum-free DMEM. (PDF 48 kb) [file 12885_2019_5803_MOESM8_ESM.pdf]

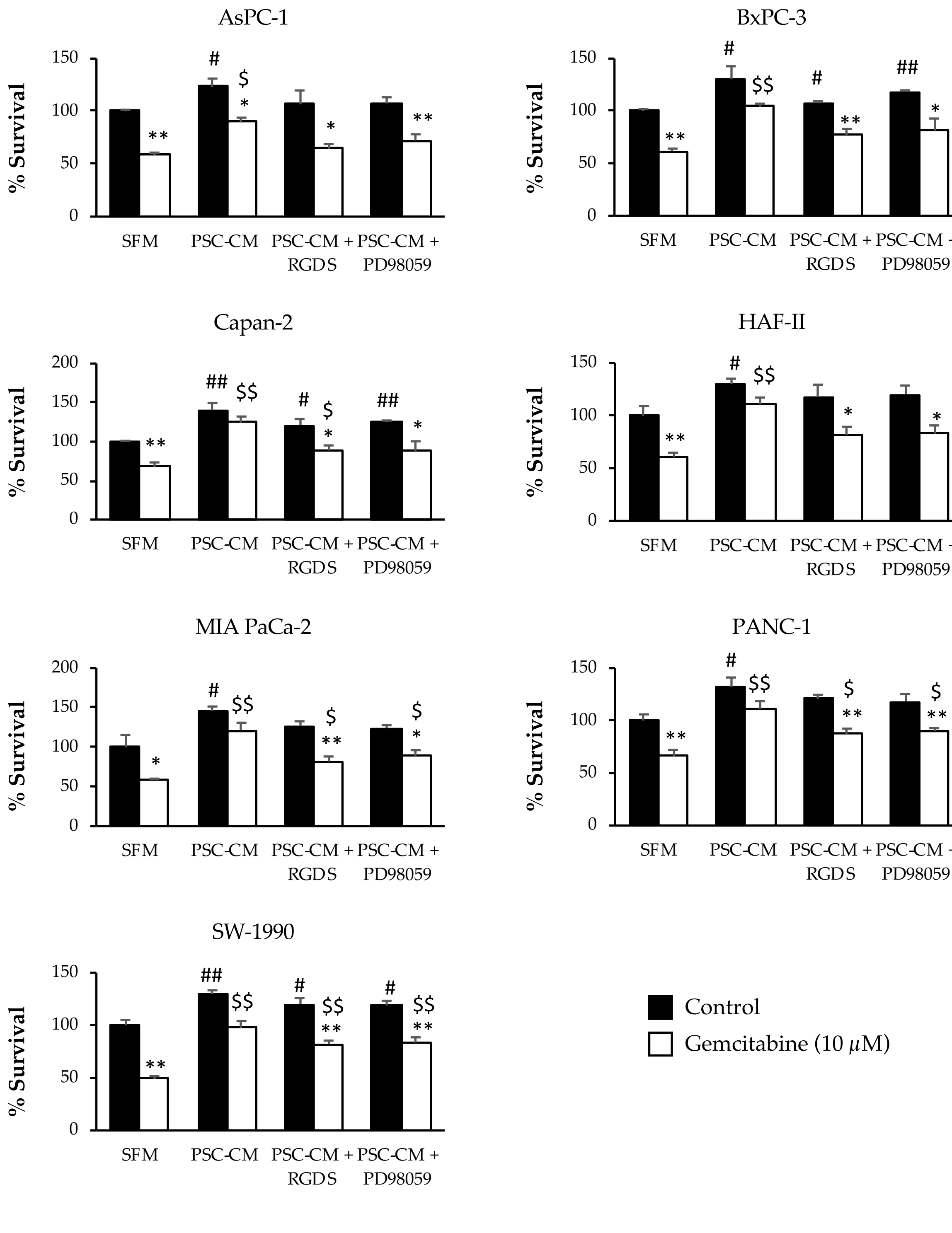

Additional File 8_Figure S5. Both FN-inhibitor (RGDS) and ERK-inhibitor (PD98059) block PSC-CM induced chemoresistance to gemcitabine. PCCs seeded on 96-well plates were incubated with SFM or PSC-CM for 24 hours and/or RGDS (20 μM) or PD98059 (20 μM) for 4 hours prior to incubation with gemcitabine (10 μM) for 48 hours. Cell viability was determined using the MTT assay. Data are the mean ± SEM of triplicate determinations. *$p<0.05$, **$p<0.01$ for control vs gemcitabine; #$p<0.05$, ##$p<0.01$ and \$$p<0.05$, \$\$$p<0.01$ for SFM vs PSC-CM/RGDS/ PD98059 in control and gemcitabine, respectively. FN, fibronectin; PSC, pancreatic stellate cell; PSC-CM, PSC-conditioned medium; SFM, serum-free DMEM.
